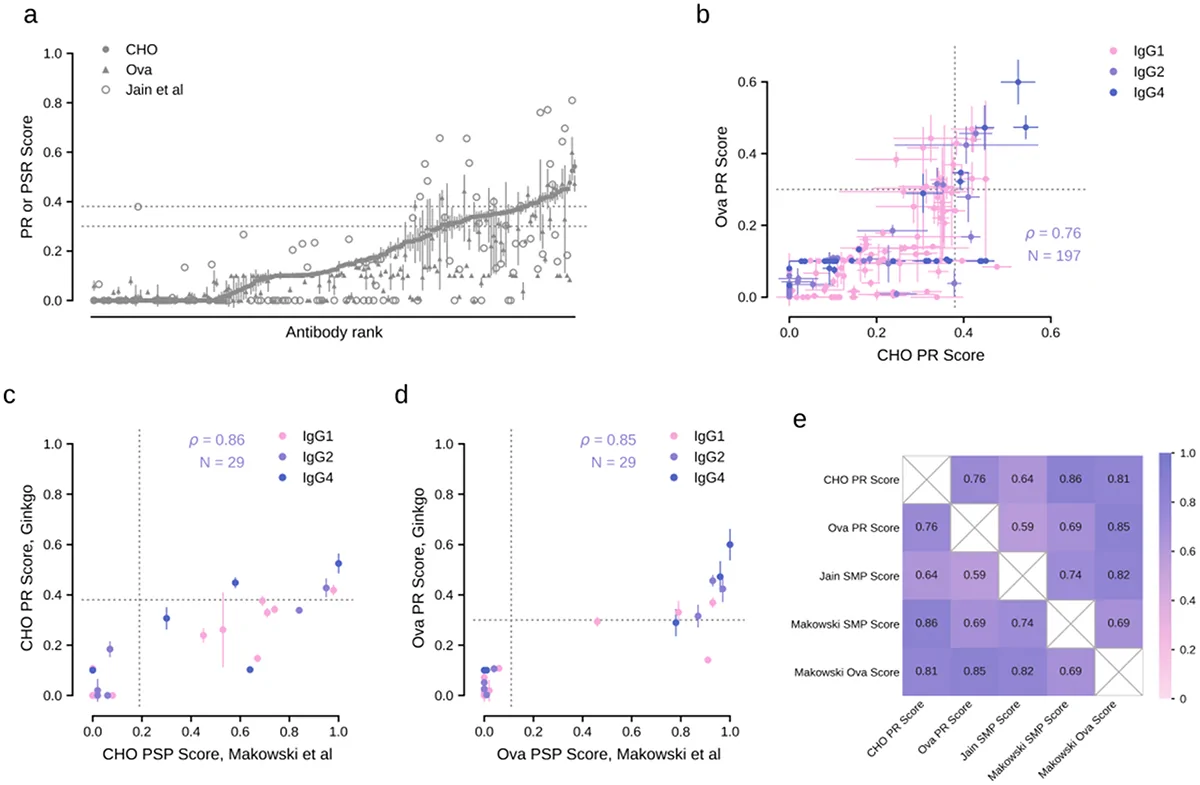# Correction

**DOI:** 10.1080/19420862.2026.2728528

**Published:** 2026-09-12

**Article title**: A high-throughput platform for biophysical antibody developability assessment to enable AI/ML model training.

**Authors**: Arsiwala, A., Bhatt, R., van Niekerk, L., Quintero-Cadena, P., Ao, X., Rosenbaum, A., Bhatt, A., Smith, A., Yang, Y., Anderson, K.C., Grippo, L., Cao, X., Cohen, R., Patel, J., Moller, J., Allen, O., Faraj, A., Nandy, A., Hocking, J., Ergun, A., Tural, B., Salvador, S., Jacobowitz, J., Schaven, K., Sherman, M., Shah, S., Tessier, P. M., & Borhani, D. W.

**Journal**: *mAbs*

**Bibliometrics**: Volume 17, Number 01

**DOI**: https://doi.org/10.1080/19420862.2025.2593055

The authors have identified a few errors in the ‘**Results and discussion**’ section, and Figures 2 and 7 have been revised. These errors arose from a data-handling error in the supplemental data file, in which the polyspecificity reagent (PSR) SMP scores reproduced from Jain et al. were transferred out of row order, so that 59 of the 135 overlapping antibodies were assigned the score belonging to a different antibody. No measurement generated in this study is affected, and the conclusions of the article are unchanged. These have now been corrected and republished in the original article.

In page number 5, second paragraph:

Under ‘***Developability properties cluster, in agreement with previous findings***’ section, the last paragraph has been updated:

“*The weak correlation we observe with polyreactivity data of Jain et al. and Shehata et al. is likely due to significant differences in methodology and assay formats*.” has been updated to “*The moderate correlation we observe with polyreactivity data of Jain et al., and the weaker correlation with that of Shehata et al., is likely due to significant differences in methodology and assay formats*.”

In page number 11, first and third paragraphs:

Under ‘***Polyreactivity assessment using CHO solubilized membrane protein and ovalbumin are comparable***’ section, the second and last paragraphs have been updated:

“*Our data generally show good agreement between CHO SMP and Ova (Figure 7b), but do not align well with the proprietary yeast-based data in the Jain dataset (Figure S2a). We believe this lack of alignment is due to two factors*.” has been updated to “*Our data generally show good agreement between CHO SMP and Ova (Figure 7b), but align only moderately with the proprietary yeast-based data in the Jain dataset (ρ = 0.64; Figure S2a). We believe this incomplete alignment is due to two factors*.”

“*Ginkgo CHO and Ova polyreactivity scores correlate well (ρ = 0.86, 0.90) with those reported by Makowski et al.^34^ for a subset of 27 common antibodies (Figure 7c, d). Of note, Makowski et al.^34^ used an IgG1 framework for all antibodies, whereas we retained the original IgG subclass. The good correlations suggest a minimal effect of the Fc region on polyreactivity, again indicating that the most polyreactive regions are predominantly located in the CDRs.^18^ A Spearman correlation heatmap (Figure 7e) emphasizes the good agreement between our data, collected using either polyreactivity reagent, and Makowski’s bead-based data, but a lack of correlation with Jain’s yeast-based method*.” has been updated to “*Ginkgo CHO and Ova polyreactivity scores correlate well (ρ = 0.86, 0.85) with those reported by Makowski et al.^34^ for a subset of 29 common antibodies (Figure 7c, d). Of note, Makowski et al.^34^ used an IgG1 framework for all antibodies, whereas we retained the original IgG subclass. The good correlations suggest a minimal effect of the Fc region on polyreactivity, again indicating that the most polyreactive regions are predominantly located in the CDRs.^18^ A Spearman correlation heatmap (Figure 7e) emphasizes the good agreement between our data, collected using either polyreactivity reagent, and Makowski’s bead-based data, and the weaker agreement with Jain’s yeast-based method*.”

**Revised Figure 2:**

**Revised Figure 7:**

The authors apologize for the error, and thank the reader who brought it to their attention.

**Figure:**
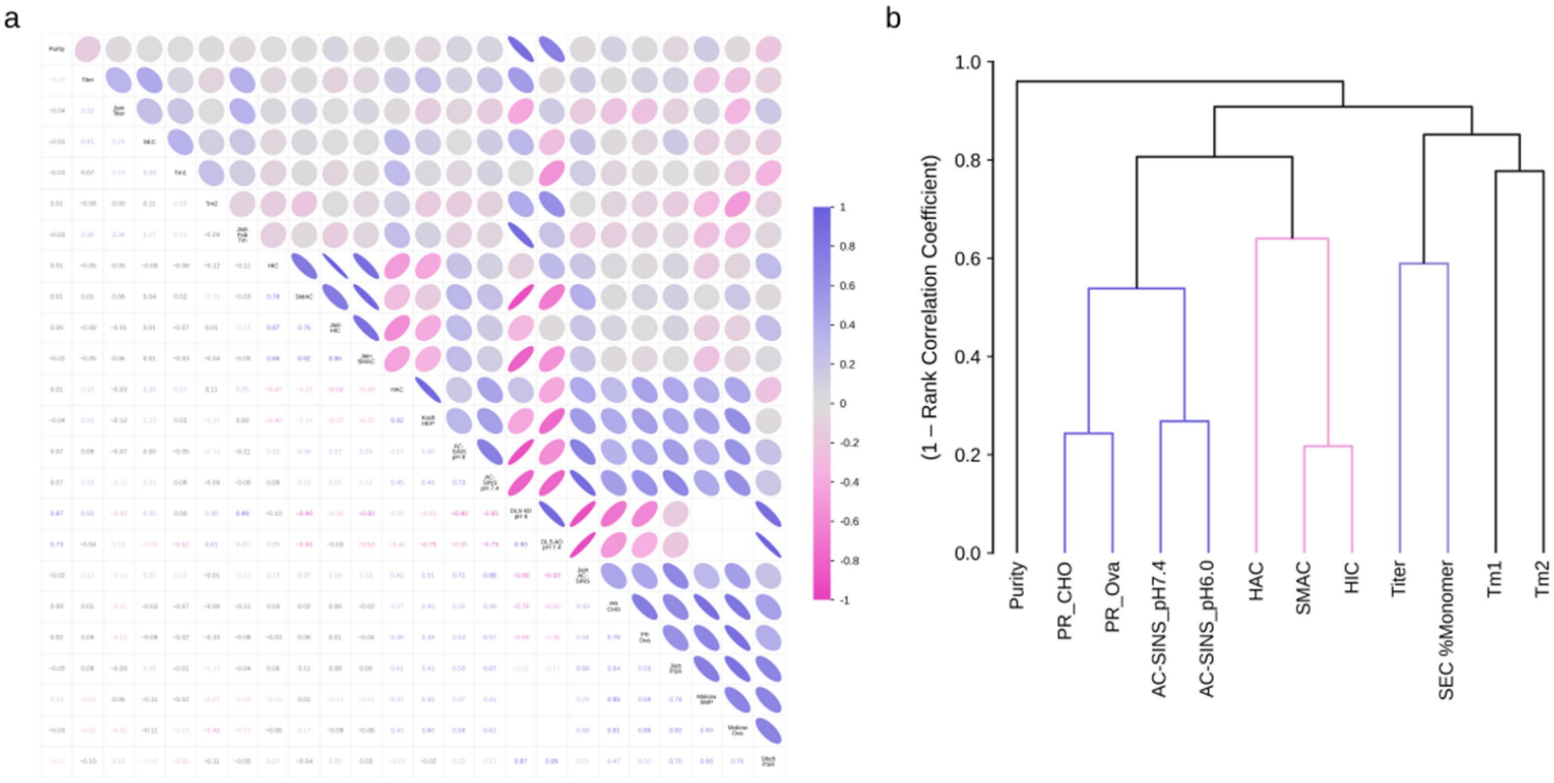

**Figure:**